# Immunologic “Cold” Squamous Cell Carcinomas of the Head and Neck Are Associated With an Unfavorable Prognosis

**DOI:** 10.3389/fmed.2021.622330

**Published:** 2021-01-27

**Authors:** Julika Ribbat-Idel, Sven Perner, Patrick Kuppler, Luise Klapper, Rosemarie Krupar, Christian Watermann, Finn-Ole Paulsen, Anne Offermann, Karl-Ludwig Bruchhage, Barbara Wollenberg, Christian Idel

**Affiliations:** ^1^Institute of Pathology, University of Luebeck and University Hospital Schleswig-Holstein, Luebeck, Germany; ^2^Pathology, Research Center Borstel, Leibniz Lung Center, Borstel, Germany; ^3^Department of Otorhinolaryngology, University of Luebeck, Luebeck, Germany; ^4^Department of Otorhinolaryngology, München rechts der Isar Technical University Munich, Munich, Germany

**Keywords:** HNSCC, FFPE, tumor microenvironment, hot, cold, excluded, p16, HPV

## Abstract

**Background:** Head and neck squamous cell carcinoma (HNSCC) represents a common cancer worldwide. Past therapeutic advances have not significantly improved HNSCC prognosis. Therefore, it is necessary to further stratify HNSCC, especially with recent advances in tumor immunology.

**Methods:** Tissue microarrays were assembled from tumor tissue samples and were complemented with comprehensive clinicopathological data of *n* = 419 patients. H&E whole slides from resection specimen (*n* = 289) were categorized according to their immune cell infiltrate as “hot,” “cold,” or “excluded.”

**Results:** Investigating tumor immune cell patterns, we found significant differences in survival rates. Immunologic “hot” and “excluded” HNSCCs are associated with better overall survival than “cold” HNSCC patients (*p* < 0.05). Interestingly, the percentage of all three patterns is nearly identical in p16 positive and negative HNSCCs.

**Conclusions:** Using a plain histological H&E approach to categorize HNSCC as being immunologic “hot,” “cold,” or “excluded” can offer a forecast of patients' prognosis and may thus aid as a potential prognostic tool in routine pathology reports. This “hot-cold-excluded” scheme needs to be applied to more HNSCC cohorts and possibly to other cancer types to determine prognostic meaning, e.g., regarding OS or DFS. Furthermore, our cohort reflects epidemiological data in the national, European, and international context. It may, therefore, be of use for future HNSCC characterization.

## Introduction

HNSCCs are the 6th most common cancer in humans (1, 2). Today the common therapies consist of surgery and/or chemoradiotherapy, which can have excruciating side effects. With the surgical approaches, patients may suffer from visible defacements, scars, and functional impairments like dysphagia or permanent voice changes. Chemoradiotherapy itself may lead to severe functional damages as well. Patients tend to suffer from dysphagia by xerostomia and necrosis, atrophy and fibrosis of the bone and the different parts of soft tissue (3).

Given these therapy-induced impairments, the prognosis is still rather poor. With an increasing tumor stage, there is a decreasing survival. For UICC stage III and IV, the 2-year survival is around 30%. Thirty to fifty percentage develop a recurrent disease (RD) which is mirrored in poor disease-free survival (DFS) (1, 4–6). Changes in therapy regimes have not improved this fate significantly for decades. The use of a neoadjuvant and also of adjuvant chemotherapy is still controversial (4, 7–9).

Especially with growing knowledge about tumor immune microenvironment (TIME), as described later in this introduction, another promising therapy option was the treatment with immune checkpoint inhibitors (ICI), with PD-L1 and PD-1 as the most prominent ICIs. PD-L1 expression on tumor cells is increased by irradiation of the tumor (10). Administering antibodies against PD-L1 and PD-1 has shown to be very successful in the treatment of several solid tumors, e.g., melanoma of the skin (11). The effects of monotherapy with these antibodies in HNSCC are, although a major improvement to current chemotherapeutic treatment standards, rather disillusioning in the overall survival (OS) (12, 13). In other publications, it has been suggested that combinational therapy might be a solution (14). However, the specific drug combination with the most promising effect on patient outcomes has yet to be found.

Clinical trials rely on biomarkers to select the most suitable patients to receive costly therapy and prevent applying potentially harmful drugs to patients that will not benefit. Thus, there is a need for research tools that can be employed for preclinical investigations. These would need to reflect typical patient features and offer a representative cancer cohort in order to test if a newly targeted antigen is actually present on tumor cells or tumor immune cells. Ideally, they could then be used to shape opinion as to whether a new drug should be considered to be passed on into the clinical trial setting.

Intratumoral immune cells have recently advanced into the focus of research groups regarding many solid tumors. Studies have been investigating the TIME in regards to their structure and contents, revealing a labyrinthian interdepending system of cells and cytokines. In several studies, researchers tried to adapt the TIME for better treatment response. Especially irradiation of tumors could induce apoptosis in cancer cells, leading to an antigen download on antigen-presenting cells by an increased MHC expression. This might be important for an increased treatment response by immune checkpoint inhibitors. On the other hand, strong irradiation can lead to lymphodepletion, so a lot of research is still needed (15). Studies of our research team showed that the composition of immune cell infiltrates contributes to improved chemoradiotherapy response in HNSCC (16).

It has been widely accepted that TIME can be categorized as being immunologic “hot” (immune cell infiltrates within the tumor), “cold” (no immune cell infiltration), or “excluded” (immune cells at tumor boundaries) (17, 18). Evaluation of immune cell parameters showed an association with survival rates and allowed prediction of response to treatments (19). In colorectal cancer, for instance, the observation of immune cell density and localization allowed a more reliable prediction of survival than the classical TNM system (20). Mainly the categories “hot” and “cold” were defined by the presence of lymphocytes, e.g., in melanomas (21). In HNSCC, the genomes of two HNSCC cohorts were analyzed for cytokine expression and the authors defined two patterns, namely high and low CD8+ T cell inflamed phenotype (22). However, genomic analyses are very expensive and also error-prone. This is why we divided the cancers as immunologically “hot,” “cold,” or “excluded” by the distribution of immune cells based on H&E analysis.

However, there are still questions to be answered such as: Do these categories exist in all solid tumor types? And can they assist in predicting patient outcome? Hypothesizing that there is a difference in the OS of HNSCC patients with different TIME patterns in primary tumors (PT), these questions are pursued in the study at hand to provide another piece in the highly complex puzzle of tumor immunology.

## Results

### Cohort Characteristics

We established a cohort of 419 HNSCC patients (22.5% female, 77.5% male) with 27.7% being p16 positive. Tissue of *n* = 4 patients was not evaluable for p16.

The majority of HNSCC PT were located in the oropharynx, larynx and oral cavity, followed by hypopharynx (Table 1). Thirteen cases were cancers of unknown primary (CUP). 48.9% of oropharynx squamous cell carcinoma (OPSCC) were p16 positive and therefore met the criterion of the newly established subtype of “p16 positive oropharynx carcinomas” according to the latest edition of TNM classification (23) and WHO classification (24). Out of these, *n* = 43 were available to TIME evaluation.

**Table 1:** Localization of primary tumors.

**Anatomical site**	**Frequency (absolute number)**
CUP^*a*^	3.1% (*n* = 13)
Hypopharynx	12.2% (*n* = 51)
Larynx	27.2% (*n* = 114)
Oral cavity	20.8% (*n*=87)
Oropharynx	33.7% (*n*=141)
Other	3.1% (*n* = 13)

a *Cancer of Unknown Primary*.

The cohort can be subdivided into two arms: patients with a local RD (25.1%) vs. patients that did not experience a cancer relapse (74.9%). Five-year survival rates ranged from 51.8 to 54.8% for oral cavity and hypopharynx cancer, respectively, to 65.0 and 67.2% for oropharynx and larynx cancer, respectively. Five-year survival rate for the whole cohort was 61.9%, for recurrent disease patients it was 55.3%. 87.6% of patients had reported nicotine abuse, whereas 43.1% had acknowledged alcohol abuse.

### TNM and UICC Stages

**Figure 1:**
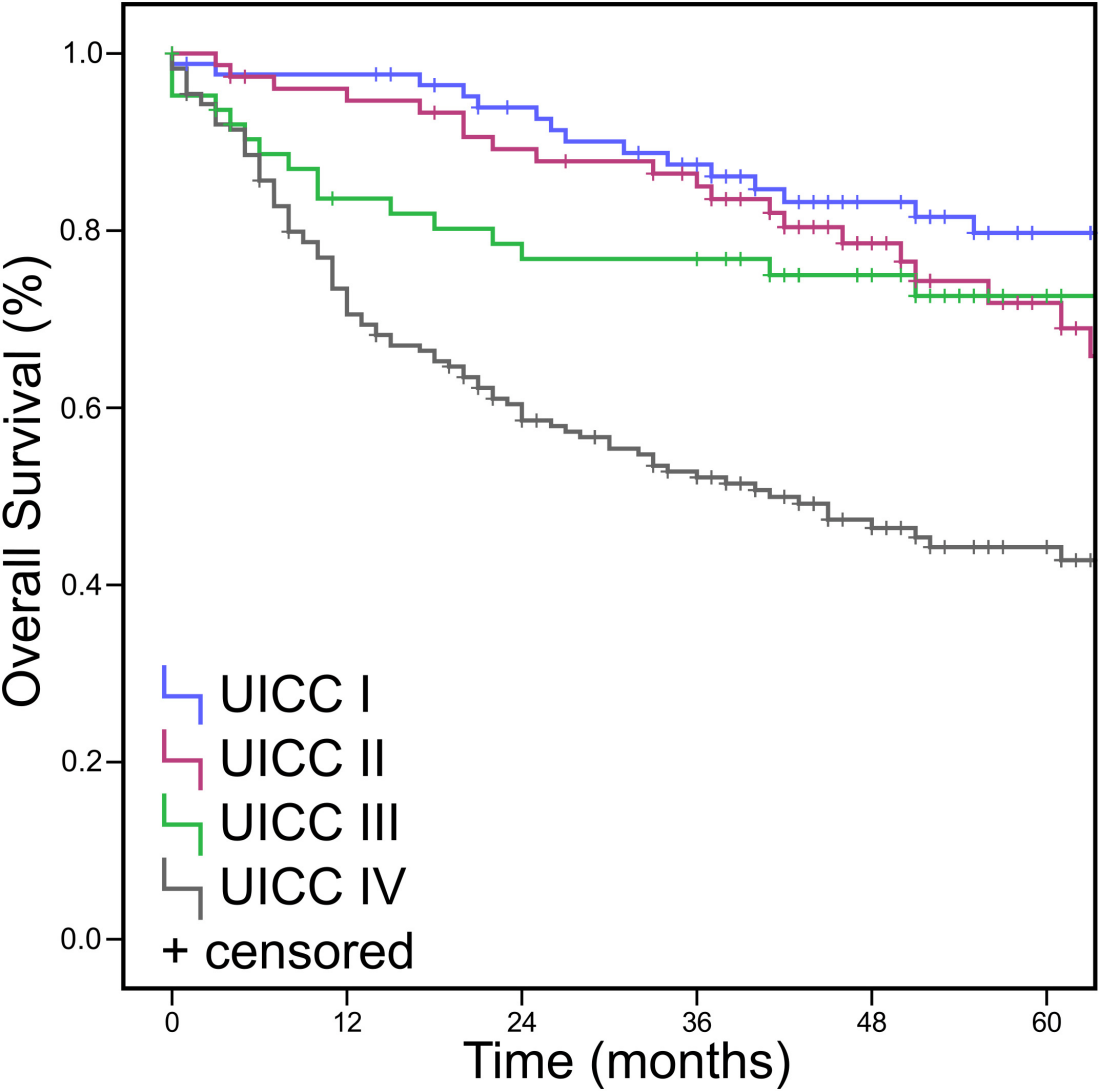
Overall Survival Data (OS, Kaplan Meier) for UICC stage. UICC stage after re-assessment according to the eighth edition of TNM classification. *p* < 0.05.

All cases were re-classified according to the 8^th^ edition of TNM classification. 50.2% of patients were classified T1/T2 and 45.4% were classified T3/4. The remaining 4.3% were TX or T0 (as in CUP). This levels with 41.1% UICC stages I/II and 58.8% UICC stages III/IV (Figure 1, Table 2). No statistically significant differences were found for “hot,” “cold,” or “excluded” tumors when assessing TIME in early vs. late UICC stages (Supplementary Figure 1).

**Table 2:** TNM and UICC stages.

**T status**	**Frequency (absolute number)**	**N/M status**	**Frequency (absolute number)**	**UICC stage**	**Frequency (absolute number)**
T0/CUP	3.6% (*n* = 15)	N0	42.6% (*n* = 176)	I	22.0% (*n* = 91)
T1	22.6% (*n* = 94)	N1	17.7% (*n* = 73)	II	19.1% (*n* = 79)
T2	27.6% (*n* = 115)	N2	25.2% (*n* =1 04)	III	15.7% (*n* = 65)
T3	25.0% (*n* = 104)	N3	14.0% (*n* = 58)	IV	43.1% (*n* = 178)
T4	20.4% (*n* = 85)	n/a	(*n* = 8)	n/a	(*n* = 6)
TX	0.7 (*n* = 3)	M0	86.6% (*n* = 362)		
n/a	(n=3)	M1	13.4% (*n* = 56)		
		n/a	(*n* = 1)		

*Re-classification according to TNM (8th edition) and UICC stage*.

PT therapy included surgery (78.8%), irradiation (59.5%) and chemotherapy (30.9%). 52% of patients suffered from nodal positive HNSCC, which was treated by surgery and irradiation in 87% and by chemotherapy in 45.2%. 13.4% developed distant metastasis (DM). 5.8% of patients reappeared with a second cancer type.

### Intratumoral Immune Cell Pattern

**Figure 2:**
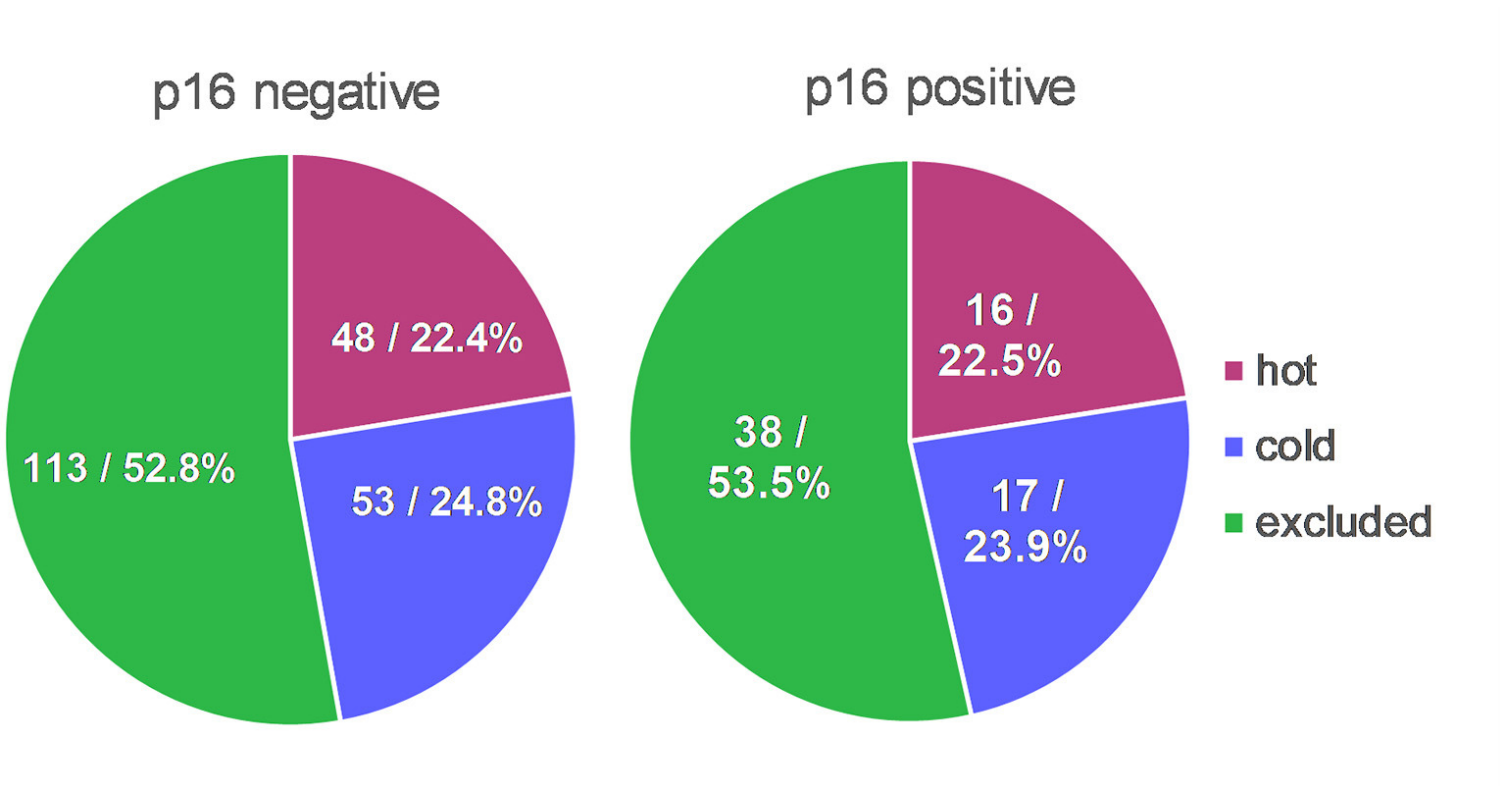
TIME in context with p16 status. Distribution of immunologic “hot,” “cold,” and “excluded” tumors were almost identical in p16 positive HNSCCs in comparison to p16 negative HNSCCs.

Two hundred eighty-nine patients received resection as first-line treatment. Their specimen of PT (*n* = 289) and RD (*n* = 42) were categorized according to their immune cell infiltrate as being “hot,” “cold,” or “excluded.” In PT, the majority showed an “excluded” phenotype (52.6%) whereas the rest was almost evenly divided as “cold” (24.2%) and “hot” (23.2%). In RDs, the vast majority was either “cold” (47.6%) or “excluded” (42.9%) with only a small portion being “hot” (9.5%). Interestingly, immunologic “hot,” “cold,” and “excluded” tumors were found in equal proportions in p16 negative and p16 positive HNSCCs (Figure 2). 75.1% of p16 positive PTs and 76.7% of p16 negative PTs were immune infiltrated (meaning either “hot” or “excluded”).

**Figure 3:**
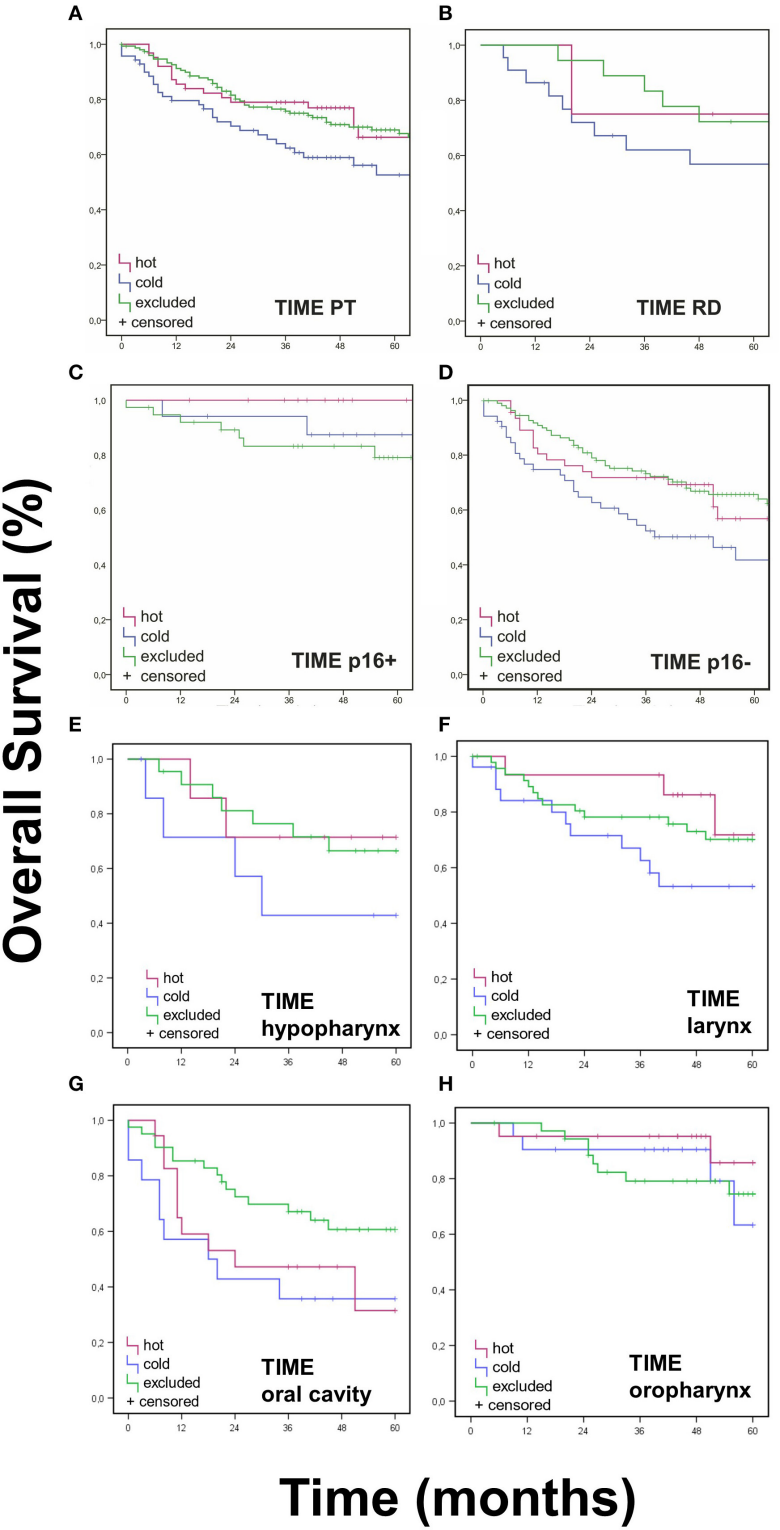
Overall Survival Data for TIME pattern. Immune cell infiltrates were classified as either “hot,” “cold,” or “excluded” tumors. **(A,B)** OS (Kaplan Meier) was statistically different for TIME assessment in PT (*p* < 0.017), but not in RD (*p* > 0.025). **(C,D)**. Neither was OS significantly different regarding p16 status (*p* > 0.017). **(G)** A better OS for “excluded” oral cavity HNSCCs was observed in comparison to “hot” and “excluded” oral cavity HNSCCs (*p* < 0.017). **(E,F,H)**. No significant differences were found for TIME in other PT locations (*p* > 0.017).

Survival data by Kaplan-Meier analysis and log-rank test showed significantly lower OS for “cold” PTs when compared to “hot” or “excluded” HNSCC (Figure 3A) after Bonferroni adjustment of the *p*-values. Accordingly, 5-year survival rates were worst for immunologic “cold” tumors.

We performed univariate and multivariate Cox regression analyses to state whether the presence or absence of immune cell infiltration of tumors (“hot” or “excluded” vs. “cold” tumors) is a significant prognostic factor for the OS of HNSCC patients and if it is independent of other prognostic factors (Supplementary Table 1). We evaluated the three immune cell patterns (“hot,” “cold,” “excluded”) and UICC stages, T stages, p16 status, sex, grading, and patient age for their prognostic value regarding OS of HNSCC patients. In the univariate analysis, it was revealed that the immune cell infiltration (Hazard Ratio (*HR*) = 0.547; *p* = 0.005), p16 expression (*HR* = 0.344; *p* = 0.001), T stage (*HR* = 1.927; *p* = 0.001), and UICC stage (*HR* = 2.212; *p* < 0.001) were significant prognostic factors for the OS. The multivariate analysis determined the immune cell infiltration pattern (*HR* = 0.527; *p* = 0.003) and the p16 expression (*HR* = 0.353; *p* = 0.001) as independent prognostic factors for the OS of HNSCC patients.

**Figure 4:**
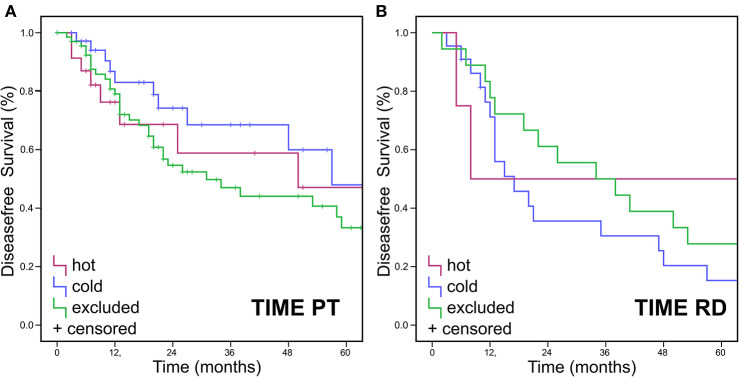
Disease free Survival Data for TIME pattern. Immune cell infiltrates were classified as either “hot,” “cold,” or “excluded” tumors. DFS (Kaplan Meier) showed no statistically significant differences for DFS in **(A)** PT or **(B)** RD.

No significant difference was found for OS when assessing TIME in RD (*p* > 0.05) (Figure 3B) and in TIME in regards to p16 status (*p* > 0.025 and *p* > 0.016, respectively) (Figures 3C,D). Neither were there significant differences in DFS for TIME in PT or RD (Figure 4).

Furthermore, no significant difference was found when observing TIME in different PT locations. In the hypopharynx, larynx, and oral cavity, roughly 55% were “excluded,” about 20% were “hot” and circa 25% were “cold” (Supplementary Figure 2). When analyzing OS in different PT locations, we found a better OS for “excluded” oral cavity HNSCCs in comparison to “hot” and “excluded” oral cavity HNSCCs (*p* = 0.048, Figure 3G). No significant differences were found for TIME in other PT locations (Figures 3E–H).

We compared OS for resected PT in combination with different therapy regimes. We found that “cold” tumor show a worse OS than inflamed (i.e., “hot” plus “excluded”) HNSCCs if the primary tumor was only treated by surgery (Supplementary Figure 3A) or by a combined regimen of surgery with an adjuvant radiochemotherapy (Supplementary Figure 3C). There was no difference if the tumors were treated in a combined regimen of surgery and adjuvant irradiation without chemotherapy Supplementary Figure 3B).

## Discussion

Introducing ICI into cancer treatment dramatically changed the fate of many cancer patients. Especially in melanoma patients, there have been large improvements in DFS and OS (16, 25–27). For patients with advanced HNSCC, the prognosis is still very poor, even after the introduction of ICI therapy in HNSCC (28). As a lot of research effort is underway many clinical trial studies compete for a limited number of participants. For wise guidance of patients into clinical trials, we need preclinical tools to estimate the success of new treatment agents.

### Cohort Characteristics

We created a cohort of 419 HNSCC patients which is well-representative of tumor epidemiology and tumor properties. This cohort reflects the typical HNSCC patients' characteristics with a male to female ratio of ~3:1 (29, 30) and a mean age of 62 years for male and 63 years for female patients. We found a positive p16 status as a surrogate marker for HPV infection in 27.7% of all HNSCC tumors (~50% of all OPSCC tumors and 17% of non-oropharyngeal HNSCC) resembling other German (23.5%) and multinational (25.9%) data (31–34). Frequencies in cancer sites in German patients show the majority in the oral cavity and pharynx [79%] and a minority in the larynx [21%] which is also reflected by our cohort (29, 30). It also mirrors the epidemiological data of national and European data [EURO-CARE-5 Study (35)] regarding the 5-year survival rate of 61.9%. With 22.6% of cases being staged as T1 (vs. 25% up to 44% in Germany), our cohort seems to lack in T1 cancer patients. This is most likely because—in order to set up a tissue-based cohort—one needs a certain amount of tumor mass to construct representative TMA cores. This naturally rules out small cancer (such as many T1 diseases) that would not yield enough tumor tissue. UICC stage IV stretches from 40% in larynx cancer to 75% in pharyngeal cancer and about half the cases of other localizations (30). In our cohort, we found 43.1% of patients in UICC stage IV. This, again, may be attributed to a selection bias as our cohort is mainly based on resectable cancers that have actually been surgically removed. UICC stage IV often reflects a palliative setting where the patient may not benefit from tumor surgery and may, therefore, undergo chemotherapy, irradiation, or best supportive care instead. So less UICC stage IV tumor material might have been available in the first place as we set up the cohort.

### TIME by H&E as a Prognostic Factor

**Figure 5:**
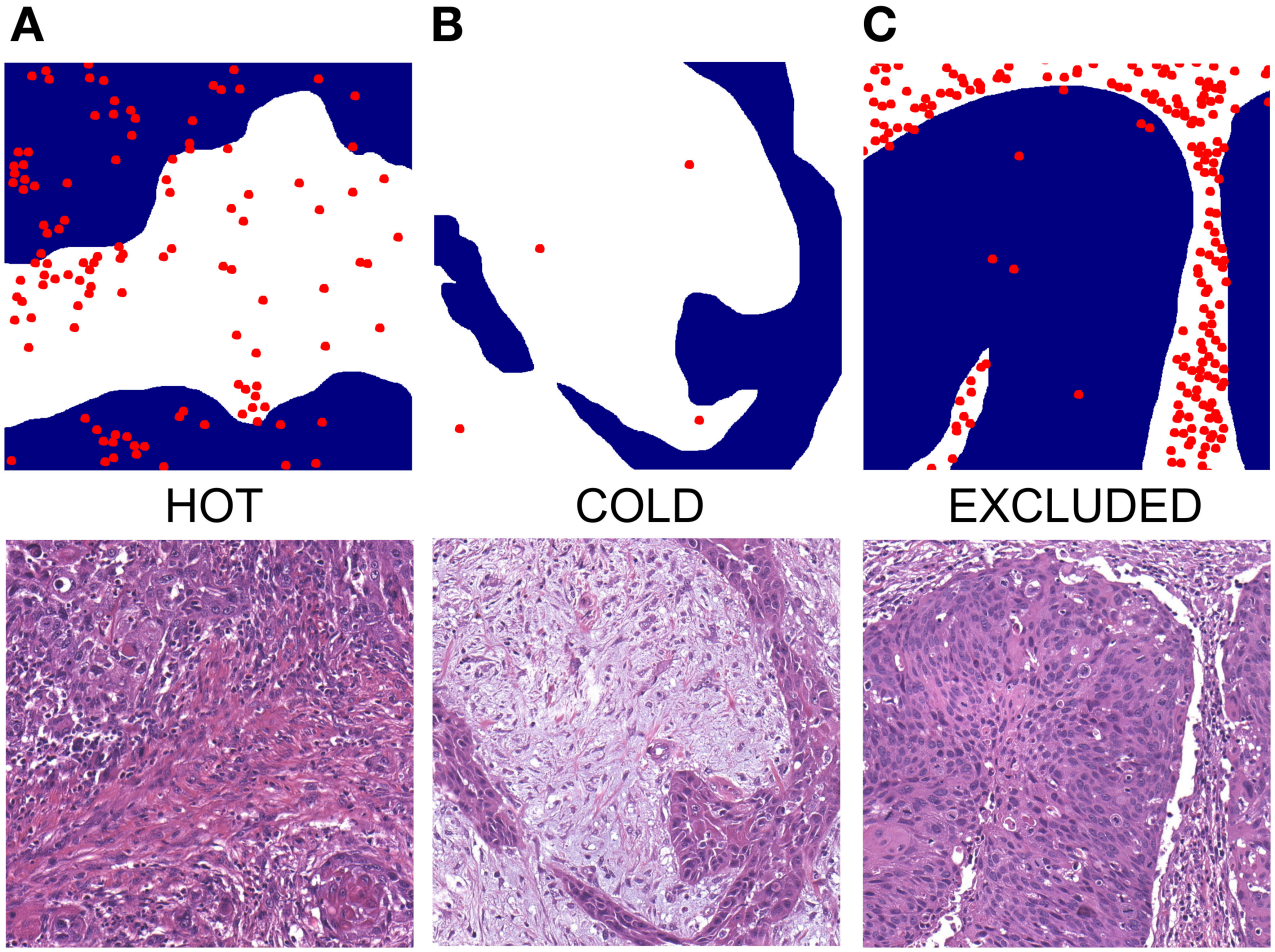
Exemplified depictions of **(A)** “hot”, **(B)** “cold,” and **(C)** “excluded” HNSCC. The upper row shows graphical TIME patterns with blue reflecting the area of cancer cells, white representing stroma, and red dots flagging the localization of immune cells. The lower row shows photomicrographs of matching typical H&E slides.

TIME in cancers can be categorized as being either “hot,” “cold,” or “excluded” by observing the distribution of immune cells in the tumor and its close neighborhood. An immunologic “hot” or “inflamed” tumor offers immune cells distributed diffusely throughout the tumor. A “cold” or “immune desert” tumor lacks immune cells whereas an “excluded” tumor shows immune cells in the desmoplastic septa of the tumor borders (18) (Figure 5, Supplementary Figure 4). It is well-known that in several tumor types the “hot” tumors are associated with a better OS (36). It has been shown that high counts of immune cells such as CD3- and CD8-positive lymphocytes within the margins of the tumor microenvironment predict a better clinical outcome in HNSCC (37). Moreover, studies suggest a crucial role of tumor-infiltrating lymphocytes (TIL) in the outcome of laryngeal squamous cell cancer concerning DFS and OS and are therefore considered of high interest in the assessment of clinical prognosis (38). We applied the categories “hot,” “cold,” or “excluded” on HNSCCs by reading routine H&E slides of resection specimens. To our knowledge, this is the first study to investigate the immune cell pattern in HNSCC by a plain histological approach.

Especially for “cold” tumors, our data showed significantly worse survival courses with worse OS and 5-year survival (Figure 3A). This is in accordance with a recent Cancer Genome Atlas (TCGA) data analysis of squamous cell carcinoma that also produced “cold” tumors to be associated with the worst prognosis (39).

Characterizing TIME as being either “hot,” “cold,” or “excluded” can easily be done by any pathologist. It is also more cost-effective and time-saving than immunohistochemical analyses as it only requires standard H&E stained slides. These categories, therefore, propose to be a cheap and intriguing prognostic marker that may be considered to be included in routine pathology reports for HNSCC. In daily routine diagnostics, Figure 5 may be used as a reference for appreciating typical TIME patterns in H&E slides. More (multi-centered) studies need to be conducted to further distinguish this “hot-cold-excluded” scheme as a potential prognostic tool.

### TIME and TNM Staging

The best and universally applied prognostic system so far was and still is grading and tumor staging by TNM/UICC. The H&E categorization in “hot,” “cold,” and “excluded” could, however, serve as a valuable amendment to it. Especially in an irresectable setting, where no pTNM staging can be established, it might be of use as an addendum to cTNM when assessed on an excisional biopsy specimen. Supposedly, it could also aid in therapeutic decision making when a cancer is right in between two stages. However, before incorporating immune cell phenotypes as predictive markers in clinical practice, further investigation of TIME with special regard to representative classification systems has yet to be conducted. We shall be eager to await validation testing being performed on other cohorts and see the “hot-cold-excluded” scheme being applied to verify prognostic meaning, e.g., regarding OS or DFS. Within early and late UICC stages, there were no statistically significant differences in OS of TIME categories (Supplementary Figure 1).

### TIME, ICI Treatment and p16

Knowing distribution patterns of immune cell types in HNSCC could now be used to elucidate if the immune type influences the outcome of an ICI treatment. Our results reveal a minority of RD to be “hot” (9.5%). So far, the only approved ICIs in Germany for the treatment of HNSCC are nivolumab and pembrolizumab. They are, however, solely approved for the treatment of RDs and not for PTs. Before that, approval studies of these drugs were mainly conducted on RD patients (28). These observations might be a part of the explanation for the worse outcome of HNSCC patients receiving ICI treatment when comparing them to other cancer types like melanoma. This hypothesis still needs further investigation. On the one hand, we must increase the patient number when analyzing TIME in RD HNSCCs to get a better estimate of the different immune types. On the other hand, we have to generate new cohorts to analyze the histology of RDs of patients before receiving an ICI treatment and compare it with the outcome of these patients under ICI treatment.

Our data show that “cold” HNSCCs show a worse OS when treated with resection only or with resection plus adjuvant chemoradiotherapy. There was no difference if the treatment was resection plus adjuvant irradiation (Supplementary Figure 3). The findings in the resection only therapy group might reflect that patients with a “cold” tumor do have a worse survival than inflamed tumors in general as this treatment is without any selective pressure of the cancer cells for therapy resistance. The different results in the groups with an adjuvant treatment are the more interesting ones because there is selective pressure for treatment resistance. The decision criteria for an adjuvant treatment are quite clear in the guidelines. If patients have lymph node metastasis without extracapsular spread (N+ ECS–) in the neck dissection they receive adjuvant irradiation only. If they have ECS in the lymph node metastasis (N+ ECS+) they receive combined adjuvant irradiation with platin-based chemotherapy. The results presented here might mean that patients suffering from “cold” N+ ECS+ HNSCC might not benefit from adjuvant combined radiochemotherapy like patients with an inflamed tumor. To get a better understanding it would be important to know if N+ ECS+ tumors that were treated with resection plus adjuvant irradiation only because of chemotherapy contraindications (e.g., kidney failure) would show a difference in OS after separating into “cold” and “inflamed” tumors. If these courses would show no difference between these two groups it might show a better response of inflamed tumors after additive chemotherapy in our presented data here. But if the “cold” N+ ECS+ tumors still had a worse OS after treatment with surgery and adjuvant irradiation only it might reflect that “cold” N+ ECS+ tumors have a worse OS in comparison to inflamed N+ ECS+ tumors in general. Our cohort limits the ability to answer these questions. We are curious to see what other (bigger? multi-center?) cohorts might show in retrospective studies when analyzing TIME in regards to treatment outcomes.

In our cohort, we could show that immunologic “hot,” “cold,” and “excluded” tumors are equally distributed in p16 positive and negative HNSCCs. This might be an explanation of why there was no difference in the subgroup analysis of HPV positive vs. negative tumors in the KEYNOTE study series leading to the FDA approval of pembrolizumab in the treatment of HNSCCs (12, 13). However, the worst OS for p16 positive HNSCC lies within the “excluded” tumors (Figure 3C) and not within the “cold” tumors like the rest of the cohort. It seems worthwhile mentioning that this finding is only a trend without statistical significance. All tumors in this cohort were analyzed for p16 expression, independent of the site of origin. p16 itself is a tumor suppressor which can also be expressed independently of HPV status (40). So the p16 expression may not be driven by HPV after all but may be caused by an upregulation of the tumor suppressor p16 due to an overexpression of different other protooncogenes. Creating subgroups within the p16 positive and negative OPSCCs and non-OPSCCs, the subgroups would get too small to yield significant results. It will be interesting to see results on this by analyses on larger cohorts.

In the subgroup analysis of PT site of origin, the tumors of the oral cavity showed a significant difference in OS (Figure 3G). Only in this subgroup, the “excluded” tumors had significantly better survival than “hot” or “cold” ones as this difference could not be found in the other sites of origin. This might be another indicator that HNSCCs are indeed a heterogeneous group of cancers rather than one entity. In another study of our group, the oral cavity HNSCCs also showed a significantly lower expression of EVI1 in comparison to HNSCCs of the oropharynx, hypopharynx, and larynx (41). The results presented here might be another hint into this direction, but whether another cytokine profile exists in oral cancers or other factors lead to better survival in “excluded” tumors of the oral cavity need further functional analysis in the future. The detachment of p16 positive OPSCCs is widely accepted and appreciated by TNM and WHO (23, 24). If more unique features of oral cavity HNSCCs are identified in the future they might meet a similar fate like p16 positive OPSCCs and be declared as a distinct entity.

HPV positive HNSCCs have been reported to be more inflamed than HPV negative ones (37). Our data showed no significant connection between p16 status and TIME. When re-grouping “hot” and “excluded” as immune infiltrated and comparing this new category to “cold” tumors we could show that immune infiltrated PTs did not track with p16 positive status (Figure 2). Therefore, TIME cannot be used to predict p16 status by observing the tumor immune cell pattern. Our cohort contains a limited number of cases with p16 positive, surgically resected OPSCC that were evaluable for TIME status. Further testing is needed on larger OPSCC cohorts which allows comprehensive analyses of the recently established p16 positive OPSCC regarding the prognostic value of the categorization into hot, cold, or excluded.

The results presented here are merely a stopover, demonstrating the usefulness of tissue cohorts. We want to use our cohort and the knowledge of the different TIME types to investigate factors leading to either a “cold,” “excluded,” or “hot” tumor to get a better understanding of cancer immunology in HNSCC.

### Conclusion

In conclusion, we have constructed a large and well-characterized tumor tissue cohort with comprehensive clinicopathological data. The cohort is well-representative of HNSCC patients and provides us with a subtle device to further investigate ICI-naïve HNSCC. Furthermore, we assessed TIME by reading the tumoral immune infiltrate pattern. We showed that the categorization of HNSCC as being either immunologic “hot,” “cold,” or “excluded” results in statistically significant differences in OS. This cheap and easy classification may, therefore, be an intriguing prognostic tool that may be considered to be applied in routine pathology reports of HNSCC, possibly even as an amendment to staging and grading. However, further evaluation is warranted and validation testing on other cohorts is needed. This “hot-cold-excluded” scheme needs to be applied to more HNSCC cohorts and possibly to other cancer types to determine prognostic meaning, e.g., regarding OS or DFS.

## Materials and Methods

### Cohort Creation

This retrospective study was conducted following the Declaration of Helsinki. It was approved by the local Ethics Committee (Ethics Committee of the University of Luebeck, AZ 16-277). The REMARK (Reporting Recommendations for Tumor Marker Prognostic Studies) checklist (42) was consulted. Planning for a target power of 80%, an effect size of 30%, and a standard deviation of 60%, we aimed for a sample size of 62 patients per group, summing up to a cohort of (at least) 186 patients.

German state law requires hospital staff to report all first-time diagnosis of cancer to regional Cancer Registries (“Krebsregister”). This is done by using the international code of disease (ICD). For our study, we researched the hospital database for ciphers for squamous cell carcinoma (ICD-O-3) and head and neck regions [ICD-10, C section (43)]. This provided us with a list of 1,266 patients. This list was double-checked for redundancies and non-squamous cell malignancies of the head and neck, e.g. lymphoma, melanoma, SNUC, etc. Those cases were excluded. We then cross-referenced this list with the clinical patient database (Agfa Orbis®) and the pathology tissue database (Nexus®) using pseudonyms. Every case was re-evaluated by a board-certified otorhinolaryngologist and a board-certified pathologist regarding the anatomical site, cancer type, and amount of available tissue. Cases were removed from the cohort if they contained too little an amount of tumor tissue or if tumor tissue had been used up during routine diagnostic procedures. Clinical data were obtained from patients' archives and Agfa Orbis® (list of clinicopathological features in Supplementary Table 2).

Then, H&E slides and paraffin blocks were drawn from the archives. After checking paraffin blocks for appropriate tumor amount the cohort was finalized with *n* = 419 patients. Patient data were anonymized. We assembled tumor tissue from PT, lymph node metastasis (LM), RD, and DM. Tissue samples were re-evaluated to classify each case according to the latest TNM classification (8th edition) and UICC stages accordingly. p16 status was determined by immunohistochemical staining of p16 (p16 CINtec ready to use kit, clone E6H4™, mouse monoclonal antibody, Roche Ventana Medical Systems, Tucson, AZ, USA). Regions of interest (ROIs) were annotated on H&E slides and paraffin blocks were matched. Three 0.1 cm cores (triplets) were punched out of every tumor to reflect heterogeneity and were then arranged in acceptor blocks as tissue microarrays (TMAs). Each TMA contained tissue triplets of 55 cancers and 5 normal mucosa samples.

### Definitions of “Hot,” “Cold”, and “Excluded”

For assessment of immune cell distribution status, we analyzed H&E whole slides from those patients from our cohort that underwent resection of PT (*n* = 289) or RD (*n* = 42). We distinguished three categories to determine TIME by reading H&E slides by the following criteria:

“Hot”—More than 2% tumor immune cells, of which more than 50% are distributed diffusely throughout the tumor, i.e. in the tumor stroma and between cancer cells.
“Excluded”—More than 2% tumor immune cells, of which more than 50% are exclusively limited to tumor stroma areas.
“Cold”—Up to 1% tumor immune cells, regardless of location.

If both “hot” and “excluded” patterns were present the one reflecting the majority was assigned. Two board-certified pathologists (JRI, RK) assessed the slides using Olympus BX50 microscope with fluorite objectives with plano-correction (Olympus Europa, Hamburg, Germany). They achieved matching results in 96% (*n* = 276). A third pathologist (SP, head of the department) was consulted to reach a consensus in the discrepant cases. Figure 5 shows a graphical depiction of these definitions and typical H&E impressions. More examples are to be found in Supplementary Figure 4. Divergent interpretations are shown in Supplementary Figure 5.

### Statistical Analyses and Software

Statistical analysis was performed using IBM SPSS Statistics 25 for Windows (IBM Corp., Armonk, NY, USA). Sixty-months OS and DFS were calculated by the Kaplan-Meier method and log-rank test for statistical significance. Individuals lost to follow-up were censored. Univariate and multivariate Cox regression analyses were performed to evaluate the association among the three immune profiles (“hot,” “cold,” “excluded”) and UICC stages, T stages, p16 status, sex, grading, and patient age. Unless multiple hypothesis testing was applied. *p* < 0.05 were considered statistically significant. For multiple hypothesis testing, we applied the Bonferroni method to adjust the *p*-value as follows. Three hypotheses were tested for PT, namely postulating a difference in the OS for different TIME patterns, postulating a difference in the OS between p16 positive and negative PTs, and postulating a difference in the DFS of patients with different TIME patterns. The Bonferroni adjusted *p*-value was *p* = 0.05/3 = 0.017. Two hypotheses were tested to RD, namely assuming a difference in the OS for different TIME patterns, and assuming a difference in the DFS for different TIME patterns. This newly adjusted *p*-value was *p* = 0.05/2 = 0.025.

We used the following software to create artwork, edit photomicrographs, and compile data visualization. Inkspace (version 0.92.4, The Inkscape Project c/o Software Freedom Conservancy, Brooklyn, NY, USA, https://inkscape.org/). Krita (version 4.2.8, Stichting Krita Foundation, Deventer, The Netherlands, https://krita.org). GIMP (version 2.10.14, The GIMP Project c/o GNOME Foundation, Orinda, CA, USA, https://www.gimp.org). Some data visualization was aided by Daniel's XL Toolbox add-in for Excel (version 7.3.4, by Daniel Kraus, Würzburg, German, www.xltoolbox.net).

## Data Availability Statement

The raw data supporting the conclusions of this article will be made available by the authors, without undue reservation.

## Ethics Statement

The studies involving human participants were reviewed and approved by Ethics Committee of the University of Luebeck. Written informed consent for participation was not required for this study in accordance with the national legislation and the institutional requirements.

## Author Contributions

CI and SP: conceptualization. JR-I, RK, and CI: methodology. AO and PK: validation. LK: formal analysis. CI: investigation. SP and BW: resources. LK and CW: data curation. JR-I: writing—original draft preparation and visualization. RK and JR-I: writing—review and editing. BW and CI: supervision. SP and K-LB: project administration. LK, CI, and F-OP: funding acquisition. All authors contributed to the article and approved the submitted version.

## Conflict of Interest

The authors declare that the research was conducted in the absence of any commercial or financial relationships that could be construed as a potential conflict of interest.

## Supplementary Material

Supplementary Figure 1 Overall survival for “hot,” “cold,” and “excluded” tumors in UICC stages I/II and UICC stages III/IV.

Supplementary Figure 2 TIME in different locations of PTs.

Supplementary Figure 3 Overall survival for “hot”, “cold”, and “excluded” tumors after different therapy approaches.

Supplementary Figure 4 Additional examples of TIME categories.

Supplementary Figure 5 Examples of divergent TIME interpretation. Both were interpreted as both “hot” and “excluded”. With help of the third observer, a consensus was reached that **(A)** classifies as “excluded” and **(B)** as “hot”.

Supplementary Table 1 Univariate and multivariate cox regression for the OS. * = p ≤ 0.05; ** = p ≤ 0.01; *** = p ≤ 0.001). Compared were the immune infiltration of the tumors (“hot” and “excluded” vs. “cold”), the T stage, UICC stage, p16 expression, grading, sex, and age of HNSCC patients.

Supplementary Table 2 List of clinicopathological features.
