# Supplementary figures and images for: Immunologic “Cold” Squamous Cell Carcinomas of the Head and Neck Are Associated With an Unfavorable Prognosis
Source: Front Med (Lausanne). 2021 Jan 27;8:622330. doi: 10.3389/fmed.2021.622330 (PMC7873597; doi:10.3389/fmed.2021.622330)

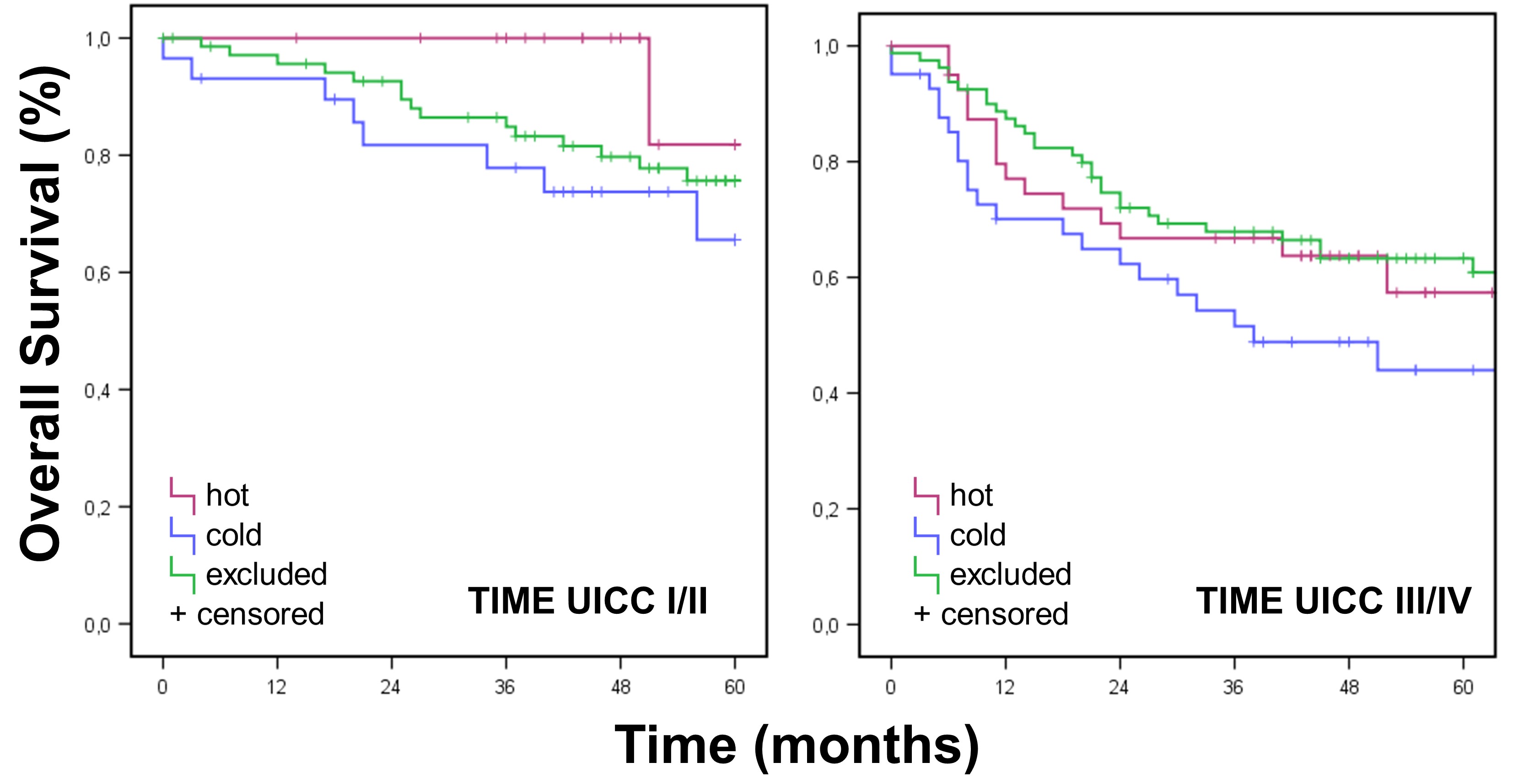

Supplement: Supplementary Figure 1 — Overall survival for “hot,” “cold,” and “excluded” tumors in UICC stages I/II and UICC stages III/IV. [file Image_1.JPEG]

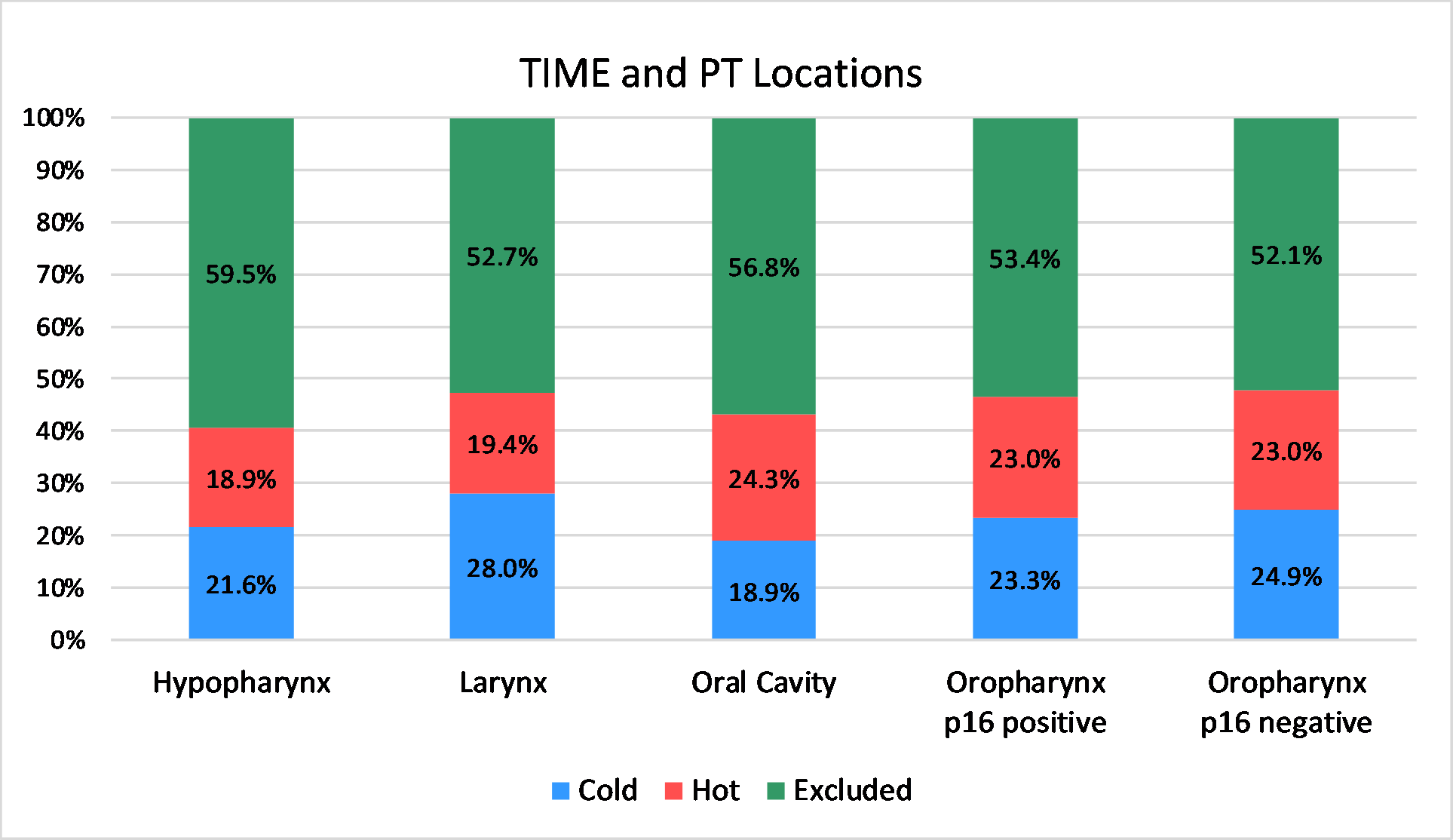

Supplement: Supplementary Figure 2 — TIME in different locations of PTs. [file Image_2.TIF]

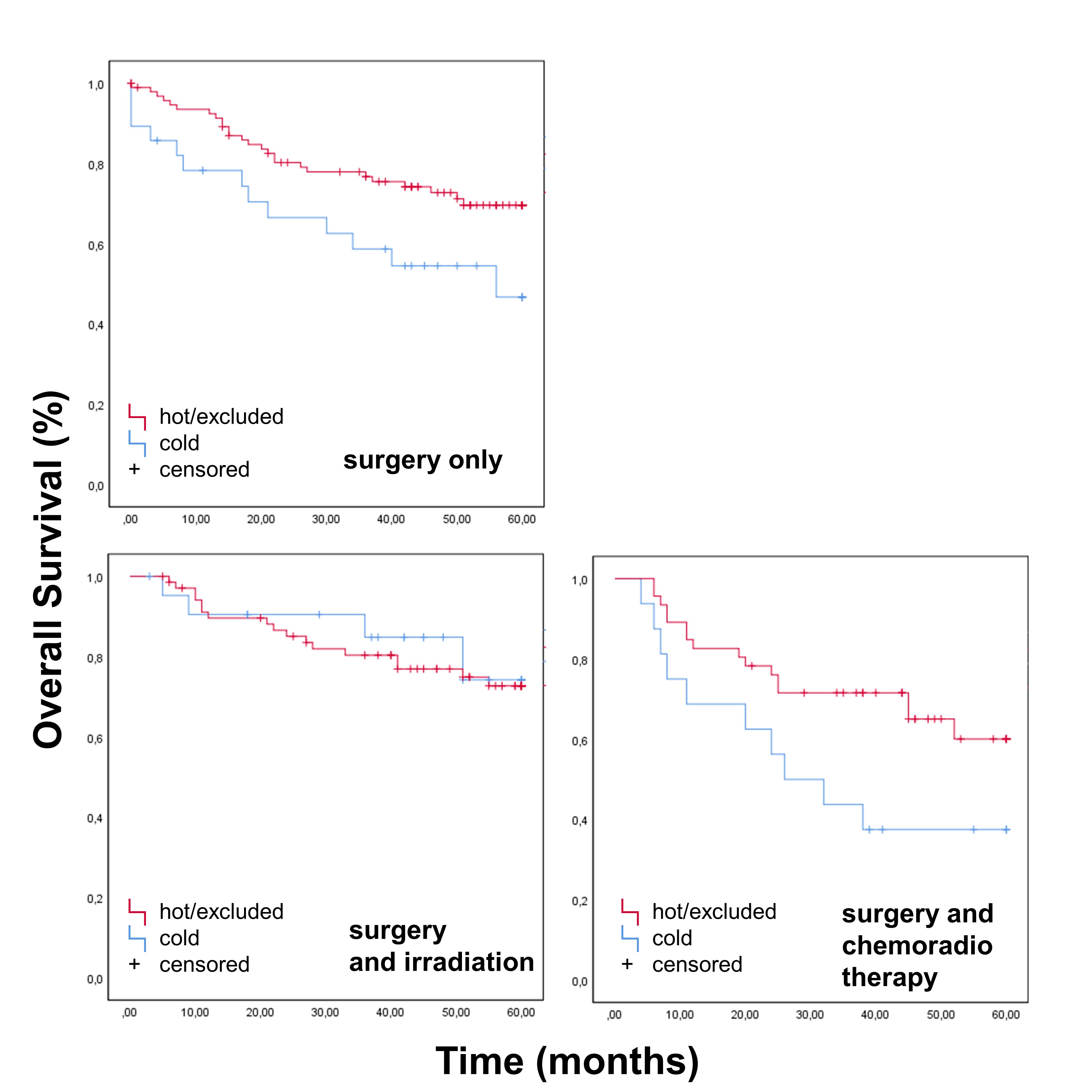

Supplement: Supplementary Figure 3 — Overall survival for “hot”, “cold”, and “excluded” tumors after different therapy approaches. [file Image_3.JPEG]

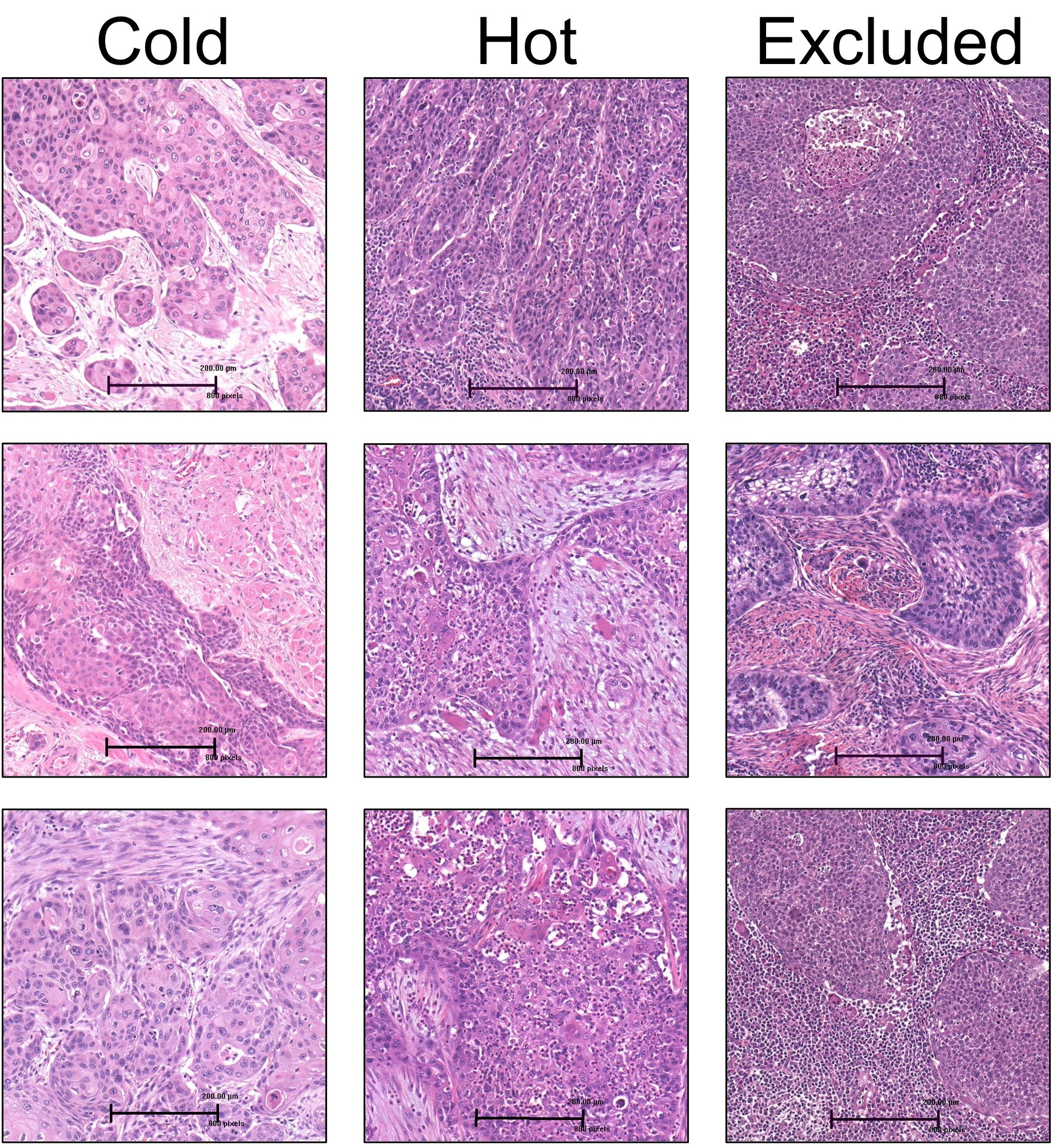

Supplement: Supplementary Figure 4 — Additional examples of TIME categories. [file Image_4.JPEG]

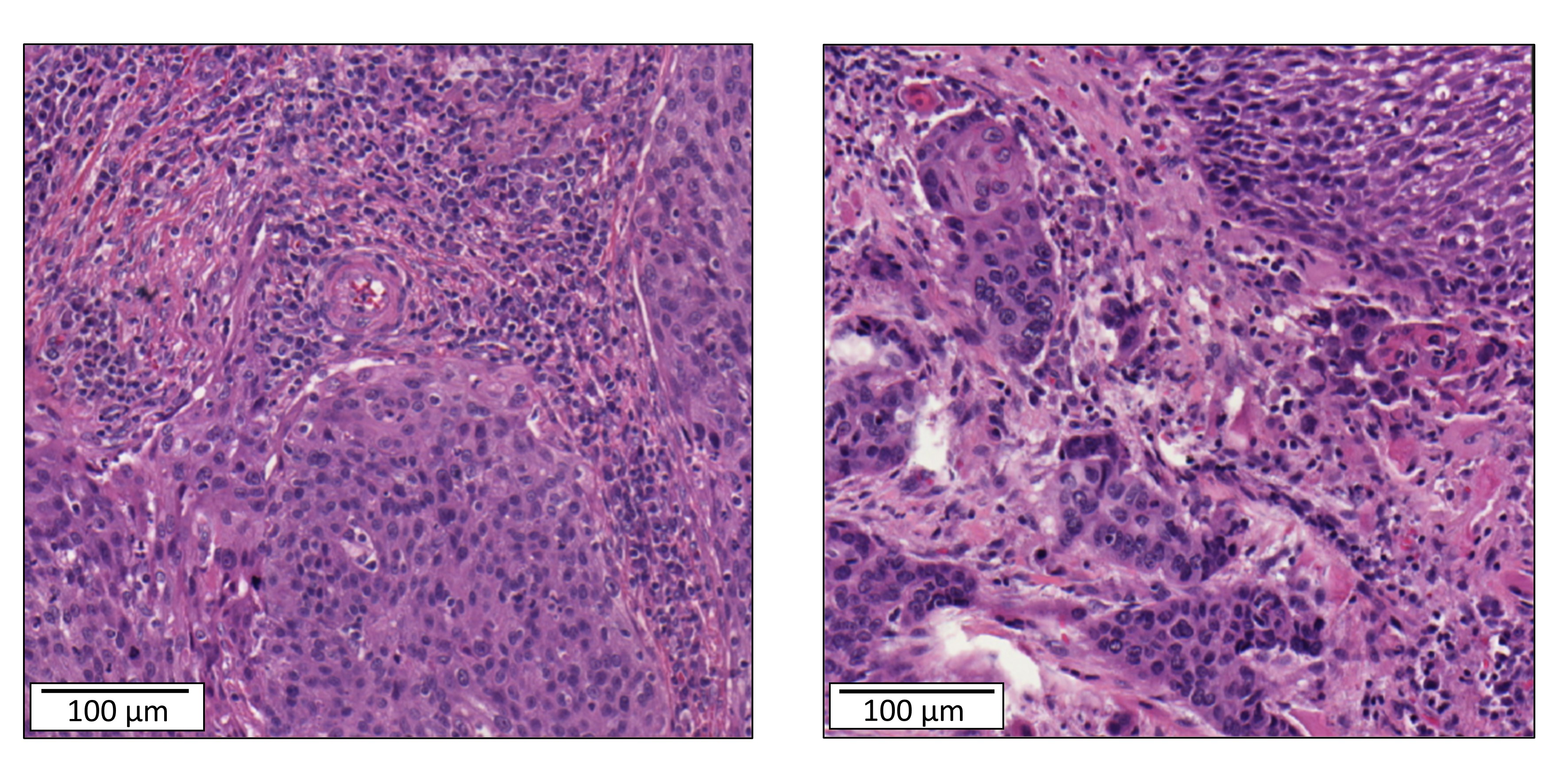

Supplement: Supplementary Figure 5 — Examples of divergent TIME interpretation. Both were interpreted as both “hot” and “excluded”. With help of the third observer, a consensus was reached that (A) classifies as “excluded” and (B) as “hot”. [file Image_5.JPEG]
