# Supplementary material for: Immunologic “Cold” Squamous Cell Carcinomas of the Head and Neck Are Associated With an Unfavorable Prognosis
Source: Front Med (Lausanne). 2021 Jan 27;8:622330. doi: 10.3389/fmed.2021.622330 (PMC7873597; doi:10.3389/fmed.2021.622330)
Supplement: Supplementary Table 1 — Univariate and multivariate cox regression for the OS. * = p ≤ 0.05; ** = p ≤ 0.01; *** = p ≤ 0.001). Compared were the immune infiltration of the tumors (“hot” and “excluded” vs. “cold”), the T stage, UICC stage, p16 expression, grading, sex, and age of HNSCC patients. [file Table_1.DOCX]

Supplementary Tables

**Supplementary Table 1**: Univariate and multivariate cox regression for the OS.
* = p ≤ 0.05; ** = p ≤ 0.01; *** = p ≤ 0.001). Compared were the immune infiltration of the tumors (“hot” and “excluded” vs. “cold”), the T stage, UICC stage, p16 expression, grading, sex, and age of HNSCC patients.

| Variable | Univariate Cox Regression | | | Multivariate Cox Regression | | |
| --- | --- | --- | --- | --- | --- | --- |
|  | p-value | Hazard Ratio | 95% Confidence Interval | p-value | Hazard Ratio | 95% Confidence Interval |
| Age | 0.165 | 1.324 | 0.891 – 1.966 |  |  |  |
| Sex | 0.556 | 1.153 | 0.718 – 1.851 |  |  |  |
| Immune infiltrate | 0.005 ** | 0.547 | 0.360 – 0.832 | 0.003 ** | 0.527 | 0.346 – 0.804 |
| T Stage | 0.001 *** | 1.927 | 1.303 – 2.849 | 0.177 | 1.446 | 0.847 – 2.467 |
| UICC Stage | < 0.001 *** | 2.212 | 1.446 – 3.384 | 0.176 | 1.497 | 0.834 – 2.687 |
| Grading | 0.354 | 1.239 | 0.788 – 1.949 |  |  |  |
| p16 expression | 0.001 *** | 0.344 | 0.188 – 0.629 | 0.001 *** | 0.353 | 0.190 – 0.657 |

**Supplementary Table 2**: List of clinicopathological features.

| Tissue | of primary tumor (if applicable |
| --- | --- |
|  | of recurrence tumor (if applicable) |
|  | of lymph node metastasis (if applicable) |
|  | of distant metastasis (if applicable) |
| Patient | Gender (F/M) |
|  | Age at first-time diagnose |
|  | Location of primary tumor |
|  | survival status (time of death, last visit to physician) |
|  | Alcohol abuse (y/n) |
|  | Nicotine abuse (y/n, pack years) |
|  | Karnofsky Index |
| Tumor | TNM, UICC – original edition when first diagnosed |
|  | TNM, UICC – re-staged according to 8th edition |
|  | grading |
|  | Recurrent disease (y/n, date) |
|  | Lymph node metastasis, distant metastasis (y/n, date) |
|  | p16 status (pos/neg) |
| Treatment | Surgery (y/n, date) |
|  | Radiation (y/n, date, dose) |
|  | Brachytherapy (y/n, date, dose) |
|  | Chemotherapy (y/n, date, dose) |
